# Pelvic Incidence: A Predictive Factor for Three-Dimensional Acetabular Orientation—A Preliminary Study

**DOI:** 10.1155/2014/594650

**Published:** 2014-03-18

**Authors:** Christophe Boulay, Gérard Bollini, Jean Legaye, Christine Tardieu, Dominique Prat-Pradal, Brigitte Chabrol, Jean-Luc Jouve, Ginette Duval-Beaupère, Jacques Pélissier

**Affiliations:** ^1^Service de Neurologie Pédiatrique, CHU Timone Enfants, 13385 Marseille, France; ^2^Département de Médecine Physique et de Réadaptation, CHU Caremeau, 30029 Nîmes, France; ^3^Laboratoire d'Anatomie, UFR de Médecine Montpellier-Nîmes, 30907 Nîmes, France; ^4^Service de Chirurgie Orthopédique Pédiatrique, CHU la Timone Enfants, 264 Rue Saint-Pierre, 13385 Marseille Cedex 05, France; ^5^Service d'Orthopédie, Clinique Universitaires UCL. de Mont-Godinne, 5530 Yvoir, Belgium; ^6^UMR 7179 CNRS, Mécanismes Adaptatifs: des Organismes aux Communautés, Pavillon d'Anatomie Comparée, Museum National Histoire Naturelle, 75005 Paris, France; ^7^INSERM U 215, CHU Raymond Poincaré, 92380 Garches, France

## Abstract

Acetabular cup orientation (inclination and anteversion) is a fundamental topic in orthopaedics and depends on pelvis tilt (positional parameter) emphasising the notion of a safe range of pelvis tilt. The hypothesis was that pelvic incidence (morphologic parameter) could yield a more accurate and reliable assessment than pelvis tilt. The aim was to find out a predictive equation of acetabular 3D orientation parameters which were determined by pelvic incidence to include in the model. The second aim was to consider the asymmetry between the right and left acetabulae. Twelve pelvic anatomic specimens were measured with an electromagnetic Fastrak system (Polhemus Society) providing 3D position of anatomical landmarks to allow measurement of acetabular and pelvic parameters. Acetabulum and pelvis data were correlated by a Spearman matrix. A robust linear regression analysis provided prediction of acetabulum axes. The orientation of each acetabulum could be predicted by the incidence. The incidence is correlated with the morphology of acetabula. The asymmetry of the acetabular roof was correlated with pelvic incidence. This study allowed analysis of relationships of acetabular orientation and pelvic incidence. Pelvic incidence (morphologic parameter) could determine the safe range of pelvis tilt (positional parameter) for an individual and not a group.

## 1. Introduction

Three-dimensional (3D) acetabular orientation is a fundamental topic in orthopaedics. A research goal in this area is to be able to predict this 3D orientation for application in surgery. Recent literature about 3D acetabular orientation focuses on 3 research areas: pelvic anatomic specimens, hip models, and clinical studies.

Definitions and measurements of 3D acetabular orientation were established by Murray [1].

Pelvic anatomic specimens showed that acetabular inclination and anteversion data used to determine spatial location of total hip arthroplasty depended on pelvis position and pelvic tilt which determined, in turn, accuracy and reliability of acetabular inclination and anteversion values [2–6]. Bonneau et al. described an innovative cadaver study for the description of the anterior and posterior acetabular rim [7].

Hip models determined acetabular cup orientation effects on range of hip rotation to improve postoperative total hip replacement stability by decreasing dislocation caused by prosthesis impingement during rotation [4]. A 3D computer-aided design model of a total hip replacement could simulate an adapted acetabular cup orientation without impingement [8].

Clinical studies, primarily related to total hip arthroplasties, also suggest that acetabular orientation and morphology depend on pelvis position and tilt, and, consequently, on patient position [9–16]. Hagio et al. [10] and Lewinnek et al. [17] emphasised the concept of a safe range of pelvis motion (pelvic tilt) for preventing hip dislocation and improving rehabilitation in postoperative activities. Lewinnek et al. described the anterior pelvic plane (APP), defined by the anterior superior spines and the pubic tubercle. The APP is commonly used as reference for positioning and postoperative evaluation of the acetabular cup in total hip arthroplasty. In an APP reference, Legaye et al. recommended that positioning of the acetabular cup in total hip arthroplasty related to anatomical parameters (sacral pelvic incidence, sacral slope, and V pubic angle) and to the global sagittal balance of the pelvispinal unit [16]. Other publications [4, 8, 12, 17–19] provided standards of acetabular inclination and anteversion within a group but not for a specific subject with respect to pelvis morphology. Different measurement procedures [1, 2, 4, 9–13, 20–23] were described to supply these data. Computer-assisted hip surgery [8, 22, 24] can determine optimal acetabular cup placement using preoperative and intraoperative planning. The computer-assisted procedure requires previous knowledge of all acetabular axes for implant performance simulation for precise final cup position relative to pelvic morphology.

Our pelvic anatomic specimen study was designed to prove that acetabular orientation is strongly correlated with pelvic parameter as suggested by previous studies [16, 25–30]. Pelvic tilt, a positional parameter depending on spine and subject positions, was demonstrated to have an influence on acetabular cup orientation [16, 17]. A morphological pelvic parameter, for example, a nonpositional parameter such as pelvic incidence [16, 26–29], is independent of subject and spine positions: pelvic incidence is defined as the angle between a line perpendicular to the sacral plate at its midpoint and a line connecting this point to the femoral head axis. Pelvic incidence is easily measured in daily practice using radiographs [26–29].

The measure of pelvic incidence is a complementary approach of the standing posture, as in scoliosis, low back pain, spondylolisthesis, spine and hip surgery, obesity, and postural impairments. The pelvic incidence, with sacral slope and pelvic tilt, determines the conditions of the principle of biomechanical economy. If these ways of adaptation between the spine and hip are unbalanced, it can improve pathological patterns on a long or short term basis. The pelvic incidence is an understanding biomechanical tool around the hip [27].

The hypothesis was that pelvic incidence could yield a more accurate and reliable assessment of acetabular cup orientation than pelvic tilt. Such a morphological pelvic parameter could determine the safe range of pelvis motion specific for an individual and not a group.

The aim was to find out a predictive equation of acetabular 3D orientation parameters (*X*, *Y*, *Z*) which were determined by pelvic incidence to include in the model. To predict acetabular orientation, pelvic incidence can be considered the independent (predictor) variable of anteversion and inclination of each acetabulum.

The second aim was to consider the asymmetry between the right and left acetabulae. The asymmetry between the acetabular morphologies, that is, right versus left femoral heads coverage, was a hypothesis according to the statement previously established for the pelvis by Boulay et al. [31]. The state of the right versus left acetabular orientation would be modified by pelvic morphology, assessed by pelvic incidence.

## 2. Material and Methods

### 2.1. Anatomical Pelvic Specimens

Twelve anatomical adult pelvic specimens with no pathological history, ages 63 to 82 years (mean 72,6 years, SD 6,25 years; 7 male and 5 female) were used for this study. The donated specimens were hand-cleaned to remove soft tissues and treated to yield dry, anatomical specimens according to a method previously described by Boulay et al. [26].

### 2.2. Anatomic Measurements

Direct measurement of the anatomical specimens was performed with an electromagnetic device (Fastrak system) which provides 3D spatial coordinate measurements. Inter- and intraobserver reliability of this method has been previously documented and was considered acceptable [32–35]. Each anatomical landmark was preliminary identified according to descriptive anatomy and 20 points, used to generate the parameters, were marked on the specimen surfaces. The points were defined by 3D spatial coordinates in relation to a common reference defined as follows.

- *Y* axis is defined by a line joining the anterior superior iliac process, oriented from right to left.
- *X* axis is perpendicular to *Y* axis, passing through the middle point of the sacral promontory, oriented frontward.
- *Z* axis is a vertical line passing through the cross product of the *Y* and *X* axes, oriented upward; the origin of the reference is defined by the intersection of *X* and *Y* axes.

Every parameter has been calculated by a common reference to compare them. The sacral plate centre was calculated mathematically by digitalisation of 8 points surrounding the upper plate of the first sacral vertebra. The middle of the bi-hip-femoral axis was equidistant from the acetabular calculated centres identified by digitalising 10 points on the concave surface of the acetabular fossa of each acetabulum.

Inter- and intraobserver reliability of the Fastrak system used was previously published [26].

### 2.3. Morphological Pelvic Parameters (Figures 1 and 2)

*Morphologic Parameters* [27–29, 36] do not vary according to pelvic orientation being constant for each pelvis and for each subject. The clinical and practical implications of these morphologic pelvic parameters are as follows. These parameters are independent (by definition) of the patient position. The values of these parameters are the same between weight bearing versus lying supine.

**Figure 1:**
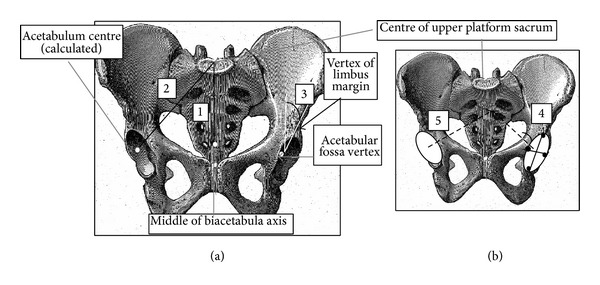
Anterior views (a, b) of pelvis. (a) 1: Thickness, 2: right beam, and 3: Hilgenreiner angle (superior lunate surface obliqueness); (b) 4: left acetabulum axis and 5: right acetabulum axis.

*Pelvic thickness *is the segment length linking the middle part of the upper plate of the first sacral vertebra (S1) and the middle of the bi-hip-femoral axis (Figure 1).

**Figure 2:**
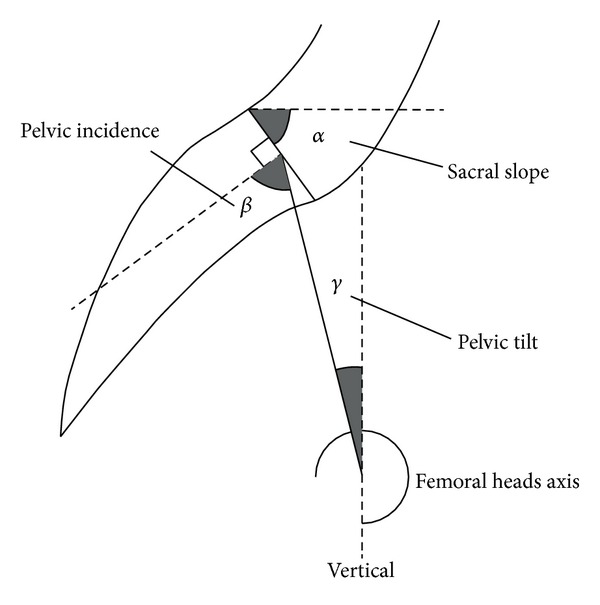
A geometric construction by complementary angles reveals that the morphological parameter incidence is the algebraic sum of the sacral slope and the pelvic tilt: Incidence = sacral slope + pelvic tilt.

*Pelvic incidence *is defined as the angle between a line perpendicular to the sacral plate at its midpoint and a line connecting this point to the femoral head axis. A large pelvic incidence corresponds to a pelvis with a horizontal sacrum and small iliac width; a small pelvic incidence indicates a pelvis with a vertical sacrum and a large iliac width (Figure 2).

**Figure 3:**
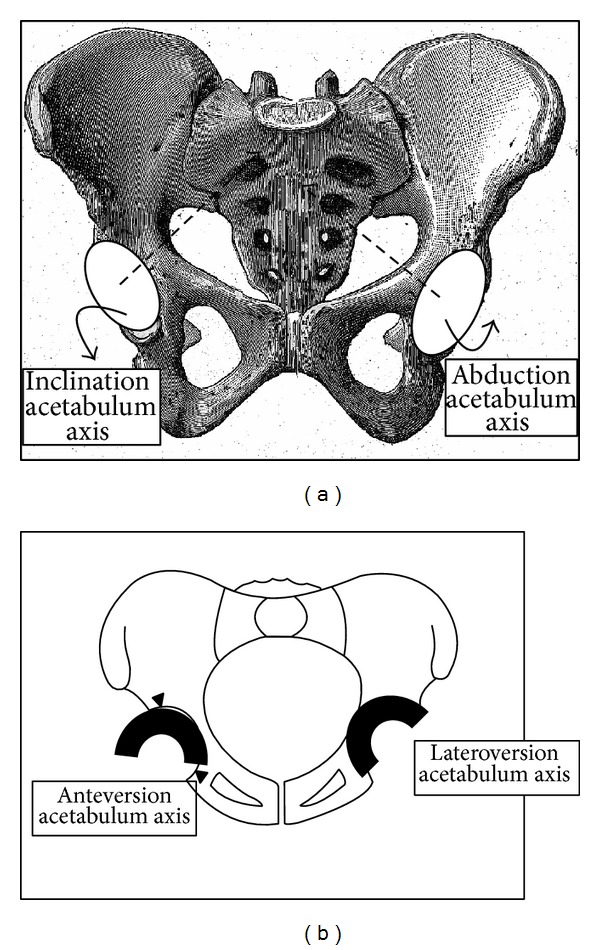
Preferential orientation of acetabula orientation with a large pelvic incidence (>62°).

**Figure 4:**
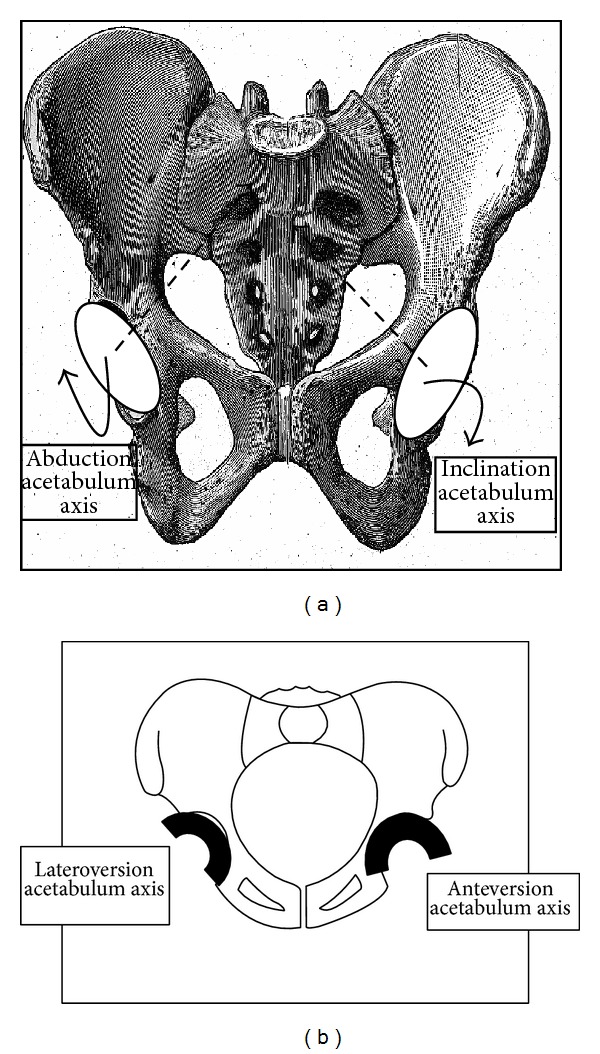
Preferential orientation of acetabula orientation with a small pelvic incidence (<44°).

*Relation between Thickness and Incidence [26]*. The pelvic incidence parameter is jointly composed by the sacrum and the iliac width. It is highly correlated to radiological measurements of the sacrum. A large pelvic incidence corresponds with a horizontal sacrum located low and anterior within the pelvis associated with small iliac width, that is, a small pelvic thickness with acetabulae close to the sacroiliac joints. Contrarily, a small pelvic incidence corresponds to a vertical sacrum located high and posterior within the pelvis and associated with a large iliac width, that is, a greater pelvic thickness with acetabulae far from the sacroiliac joints (Figures 3 and 4).

*The left beam* is the segment length linking the middle part of the S1 upper plate and centre of the left acetabulum.

*The right beam* is the segment length linking the middle part of the S1 upper plate and centre of the right acetabulum (Figure 1).

A large beam corresponds to a large iliac width and, conversely, a small beam means a short iliac width.

*Hilgenreiner angle [31, 37] *assesses femoral head coverage and is defined as the angle between a horizontal line at the top of acetabular fossa and a line connecting this point to the top of the concave surface. A large Hilgenreiner angle corresponds to better coverage of the femoral head. Similarly, a small Hilgenreiner angle means limited coverage of the femoral head (Figure 1).

*Acetabulum axis* is a straight line perpendicular to the centre of a least squared plane described by the circumference of the concave surface of the acetabulum (Figure 1).

*The direction cosines of acetabulum axis* (cos⁡*X*, cos⁡*Y*, and cos⁡*Z*) are the trigonometric cosines of the unitary vector of acetabulum axis on the references axes (*X*, *Y*, *Z*). The third cosine can always be recomputed from the other two since the sum of squares of all three always equals1: (cos *X*)^2^ + (cos *Y*)^2^ + (cos *Z*)^2^ = 1.

By definition:

- the origin of the reference axes is the acetabular calculated centre,
- the (*X*, *Y*) plane is horizontal:* anteversion* of acetabular cup if cos *Y* is more important than cos *X*,* retroversion *of acetabular cup if cos *X* is more important than cos *Y* and, on the contrary,
- the (*X*, *Z*) plane is frontal:* inclination* of acetabular cup if cos *Z* is more important than cos *X* and, on the contrary,* abduction* of acetabular cup if cos *X* is more important than cos *Z*.

*Acetabular incidence *is the angle between the pelvic thickness and the bisection of the acetabula axes (Figure 1).

*Positional Parameters* [27–29, 36] vary according to pelvic position of in spaceand are subject dependent (Figure 2).

*Sacral slope *isdefined as the angle between a sacral plate and the horizontal line. A vertical sacrum is described by a low sacral slope value and a horizontal sacrum by a high value sacral slope (Figure 2).

*Pelvic tilt *is defined by (1) a line through midpoint of the sacral plate and midpoint of the femoral head axes and (2) the vertical line through the midpoint of the femoral head axis. Pelvic tilt is positive when the sacral plate is behind the hip and negative when it is in front of it (Figure 2).

### 2.4. Relation between Positional and Morphologic Parameters [27–29, 36]

A geometric construction using complementary angles showed the morphological parameter incidence is the algebraic sum of the sacral slope and the pelvic tilt: incidence = sacral slope + pelvic tilt (Figure 2).

### 2.5. Statistical Analysis

Data normalization was tested by Kolmogorov-Smirnov normality tests.

Taking into account the small sample size (*n* < 30), a Spearman correlation matrix was used to assess the extent of the relation between acetabular morphology (i.e., right and left Hilgenreiner angles) and pelvis morphology (angle: pelvic incidence; distances: thickness, right and left beams). Each metric parameter was correlated with its real value expressed in a dimensional unit (millimetre) but also in a dimensionless unit. The choice of the dimensionless [16] factor was the homologous acetabular diameter for the paired parameter and the left and right acetabular mean for the unpaired parameter. This value is proved by the influence of the acetabular diameter on hip biomechanics and pelvis morphology [6, 15].

A multivariate selection algorithm (MacHenry Algorithm) was run using the acetabular axes (*X*, *Y*, *Z*) as the dependent (or predicted) variable and a single (because of the small sample) pelvic parameter as the independent (or predictor) variable.

To establish a mathematical model to predict the acetabular axes (*X*, *Y*, *Z*), a robust linear regression analysis was used because the sample is small (*n* < 30). This method attenuates the leverage of influential outliers which might bias the prediction. This regression analysis lessens the influence of outliers by identifying these outliers and minimising their impact on the coefficient estimates. A *P* value of 0.05 was regarded to be significant.

## 3. Results 

### 3.1. Distribution of the Data

The Kolmogorov-Smirnov normality test accepted the normality for all pelvic parameters.

### 3.2. Correlation between Acetabular and Pelvic Morphology

In the dimensional unit (Table 1), the correlations between Hilgenreiner angles and the posterior acetabular area (thickness, left and right beams) were always not statistically significant.

In the dimensionless unit (Table 2), only thickness was statistically positively significant with the right Hilgenreiner angle (*r*_*s*_ = 0.69, *P* = 0.01) and negatively with the left (*r*_*s*_ = −0.56, *P* = 0.05). The negative correlation between thickness and incidence (*r*_*s*_ = −0.66, *P* = 0.01) was significant.

The left and right Hilgenreiner angles were negatively correlated: *r*_*s*_ = −0.636 and *P* = 0.026.

### 3.3. Predictive Equations of Acetabular Axes

The linear regression equations of each (*X*, *Y*, *Z*) acetabular axis (Table 3) provided established a mathematical model to predict the acetabulum axis (*X*, *Y*, *Z*) with a single predictor variable: pelvic incidence. Consider the following.

 
*Cos*⁡*Z* left acetabulum axis = 0.790657 − 2.888895E-03 ∗ pelvic_incidence.
 
*Cos*⁡*X* left acetabulum axis = −0.65069349 − 1.503430647E-03 ∗ pelvic_incidence.
 
*Cos*⁡*Y* left acetabulum axis = 0.452038347 − 3.428956031E-03 ∗ pelvic_incidence.
 
*Cos*⁡*X* right acetabulum axis = 0.783363368 − 6.70547841E-04 ∗ pelvic_incidence.
 
*Cos*⁡*Y* right acetabulum axis = 0.497428597 − 3.98299646E-03 ∗ pelvic_incidence.
 
*Cos*⁡*Z* right acetabulum axis = 0.430076926 + 3.186066111E-03 ∗ pelvic_incidence.

For each model, check model adequacy is achieved (linearity, normality, and independence of residuals). The residual (difference between measured and predicted variable) for each (*X*, *Y*, *Z*) acetabular axis, in degree, was very minor (Table 4). The accuracy of each model is assessed by the confidence limits and the difference between measured (or theoretical) value minus predicted value.

The predicted (*X*, *Y*, *Z*) acetabular axis accuracy of each model was assessed by confidence limits, which were very minor in degree (Table 4).

## 4. Discussion

The first assumption of our study was to prove that acetabular morphology is correlated with a morphologic pelvic parameter, the pelvic incidence, whose value is constant and independent of pelvis space, position, and dimension, justifying the choice of a dimensionless unit.

The second study assumption was to provide a morphological pelvic parameter, the pelvic incidence, able to predict a safe range of pelvis motion and pelvic tilt, which governs acetabular cup orientation.

The method, validated and applied to anatomic specimens, is reliable in studying normal and abnormal groups [26]. Although all parameters had a normal distribution, the small sample size (*n* < 30) affords only preliminary conclusions.

Pelvic incidence is the morphologic parameter that validated these two assumptions. Pelvic incidence [26, 28] is negatively correlated with dimension and dimensionless thickness: a large pelvic incidence [27] corresponds to a pelvis with a small iliac width, that is, a small thickness (Figure 3). Conversely, a small pelvic incidence means a pelvis with a large iliac width, that is, a great thickness (Figure 4). Thickness, in a dimensionless unit, is correlated with the acetabular morphology that determined the femoral head coverage. Right versus left Hilgenreiner angles were not influenced the same way. A pelvis with a small incidence [27] (<44°) (Figure 4), that is, with a large thickness, is described with a great right Hilgenreiner angle, that is, high right femoral head coverage and, on the contrary, with a small left Hilgenreiner angle, that is, low left femoral head coverage. A pelvis with a large incidence [27] (>62°) (Figure 3), that is, with a small thickness, is described with a small right Hilgenreiner angle, that is, low right femoral head coverage and, conversely, with a large left Hilgenreiner angle, that is, high left femoral head coverage. These facts were verified by the negative correlation between the left versus right Hilgenreiner angles. The method is justified on one hand by the choice of a dimensionless unit and on the other hand by the choice of the dimensionless factor (acetabular diameter).

Duval-Beaupere et al. [28] described the relationship between spinal alignment (lordosis, kyphosis), positional pelvic parameters (whose values vary, by definition, with position of the subject), and pelvis morphology (whose values are constant for each subject and independent of subject position) (Figure 2).

Any change in one parameter induces a change in the other, except for pelvic incidence. In fact, sacral slope influences the suprapelvic level (lordosis, kyphosis) but pelvic tilt affects the infrapelvis level, that is, the hip-femoral joints in different subject positions. The ability of the functional spine-pelvis unit to seek and maintain sagittal alignment in different positions depends on the morphological parameter incidence and on the distribution of the other positional parameters. The range of motion of these positional parameters (spinal and pelvic) determined the adaptability of the lumbar and dorsal curves and the pelvis position (pelvic tilt and sacral slope) [27–29, 36]. Thus the potential of adaptation or range of motion of the above spinal curves is associated with the one of pelvic positional parameters according to the relation “incidence (morphology) = sacral slope (positional) + pelvic tilting (positional).”

We consider that the sacral slope is maximal in the standing position and minimal while sitting. Between these positions, the range of pelvis tilt is maximum in normal subjects. Pelvic incidence being the algebraic sum of the sacral slope and the pelvic tilt, it determines the range of motion of pelvic tilt that varies among the subject positions and explains the large variability of this parameter. The measure of incidence determined, for an individual in particularly, its own range of motion of pelvic tilt. For example, with a large incidence, for example, 62°, the range of pelvic tilt is very large, varying from 62° to 0° or even negative values, according to subject position. In contrast, with a small pelvic incidence, for example, 44°, the range of pelvic tilt is smaller, varying from 44° to 0° or even negative values, according to subject position. The measure of pelvic incidence explained the data, described by Hagio et al. [10] and Lewinnek et al. [17], of a safe range of pelvis motion (pelvic tilt) for preventing hip dislocation and improving rehabilitation in postoperative activities. In daily practice, pelvic incidence which can be measured on radiographic images appears more relevant and reliable than pelvic tilt as the main factor of acetabulum orientation.

To predict acetabular orientation, pelvic incidence can be considered the independent (predictor) variable of anteversion and inclination of each acetabulum for computer-assisted surgery. The state of the right versus left acetabular orientation is modified by pelvic morphology, assessed by pelvic incidence (Figures 3 and 4). When the pelvic incidence is small (<44°), inclination and anteversion of the left acetabulum are more pronounced in contrast to the abduction and retroversion predominant for the right acetabulum (Figure 4). When the pelvic incidence is large (>62°), the retroversion and abduction are great for the left acetabulum and the anteversion and inclination are more pronounced for the right acetabulum (Figure 3).

The right and left acetabulae were considered not to be symmetric. The asymmetry between the acetabular morphologies, that is, right versus left femoral heads coverage, confirms this notion previously established for the pelvis (iliac wing) by Boulay et al. [31]. A spiral aspect is present in the pelvis with the upper iliac wings rotating clockwise and the lower part rotating counterclockwise. The cause of this asymmetry is unknown but may be a reflection of extremity laterality dominance or due to gait variability.

Moreover these clinical data could be used in studies on models of hip prostheses. Thus Mattei et al. [38] reported a review of both lubrification and wear models focusing on their main characteristics of the hip implant tribology: they underlined the interest that new advanced models including both aspects could be helpful. With our study, we could add that pelvic incidence could be included in these advanced models in order to obtain not only a 3D acetabular orientation but also a global pelvic morphology. Thus the awareness of pelvic incidence, that is, also the asymmetry between acetabular orientations, could increase the tribological performance of the artificial hip joint.

Jun and Choi [39] developed a software system that designed a patient-specific hip implant by investigating the anatomical 3D geometry parameters of the patient's femur. Then a custom-made femoral implant based upon the extracted parameters is created. Taking into account patient's pelvic incidence, that is, also the asymmetry between acetabular morphologies, a custom-made hip implant could be obtained in the best-fit to a patient preventing hip dislocation and improving rehabilitation after total hip replacement.

## 5. Conclusions

This preliminary data found in this study needs to be confirmed using a larger sample (>30) size. This study emphasised the relevance and the necessity to study the relationships between the spine-pelvis alignment, the pelvis morphology and the acetabular orientation in different postures of the subject (standing, sitting, and lying) and the asymmetry.

Thus these preliminary data underlined the interest to measure pelvic incidence in clinical practice. Pelvic incidence quantified more especially the concept of a physiological (for each patient) range of pelvis motion (described, initially, by Hagio et al. [10] et Lewinnek et al. [17]) with the sacral slope and pelvic tilt. The understanding of the interaction between these parameters could improve the preventing hip dislocation and the rehabilitation (before and after surgery), strengthening and stretching programs of rectus femoris, iliopsoas, or hamstrings and, also, the hip internal and external rotators muscles.

Pelvic incidence is a 3D morphological parameter that determines the conditions of the principle of biomechanical economy between spine and femur: it is an anatomical key parameter for clinicians and researchers (e.g., hip model modulated by the pelvic incidence value).

## Figures and Tables

**Table 1:** Spearman correlation section: acetabular and pelvic morphology (dimensional unit).

Parameter	Thickness (mm)	Right beam (mm)	Right Hilgenreiner angle (°)	Left beam (mm)	Left Hilgenreiner angle (°)
Thickness	—	0.937	0.48	0.790	−0.08
	—	0.000007	0.11	0.002	0.81
	—	S***	NS	S**	NS

Right beam	0.937^¤^	—	0.4	0.63	0.01
	0.000007^§^	—	0.2	0.03	0.97
	S^∗∗∗&^	—	NS	S*	NS

Right Hilgenreiner angle	0.48	0.4	—	0.46	−0.64
	0.11	0.2	—	0.13	0.03
	NS	NS	—	NS	S*

Left beam	0.790	0.63	0.46	—	−0.11
	0.002	0.03	0.13	—	0.73
	S**	S*	NS	—	NS

Left Hilgenreiner angle	−0.08	0.01	−0.64	−0.11	—
	0.81	0.97	0.03	0.73	—
	NS	NS	S*	NS	—

^¤^Upper column: correlation coefficient; ^§^middle column: *P* value; ^&^lower column: significant.

*P < 0.05; **0.001 < *P* < 0.01; ***P < 0.001, alpha = 5%.

**Table 2:** Spearman correlation section: acetabular and pelvic morphology (dimensionless unit).

Parameter	Thickness dimensionless	Right beam dimensionless	Right Hilgenreiner angle (°)	Left beam dimensionless	Left Hilgenreiner angle (°)
Thickness	—	0.94	0.69	0.87	−0.56
	—	0.000006	0.01	0.00028	0.05
	—	S***	S**	S***	S*

Right beam	0.94^¤^	—	0.54	0.83	−0.41
	0.000006^§^	—	0.07	0.00095	0.19
	S^∗∗∗&^	—	NS	S***	NS

Right Hilgenreiner angle	0.69	0.54	—	0.50	−0.64
	0.01	0.07	—	0.1	0.03
	S**	NS	—	NS	S*

Left beam	0.87	0.83	0.50	—	−0.45
	0.00028	0.00095	0.10	—	0.14
	S***	S***	NS	—	NS

Left Hilgenreiner angle	−0.56	−0.41	−0.64	−0.45	—
	0.05	0.19	0.03	0.14	—
	S*	NS	S*	NS	—

^¤^Upper column: correlation coefficient; ^§^middle column: *P* value; ^&^lower column: significant.

*P < 0.05; **0.001 < *P* < 0.01; ***P < 0.001; alpha = 5%.

**Table 3:** The regression equations: direction cosines of acetabulum axis (*X*, *Y*, *Z*).

Predicted (dependent) variable	Predictor (independent) variable	Regression coefficient	*P*-level	Standardized coefficient
*X* right acetabulum axis (cos⁡X_r)	Intercept	1.100105		0.0
	(cos⁡Z_r)	−0.6262985	0.0007	−0.857
	*R*-squared	0.73		

*Y* right acetabulum axis (cos⁡Y_r)	Intercept	−2.764875*E* − 02		0.0
	(cos⁡Y_l)	1.161577	0.006	0.76
	*R*-squared	0.58		

*Z* right acetabulum axis (cos⁡Z_r)	Intercept	1.167902		0.0
	(cos⁡X_r)	−0.7642675	0.0006	−0.85
	*R*-squared	0.739		

*X* left acetabulum axis (cos⁡X_l)	Intercept	−1.062165		0.0
	(cos⁡Z_l)	0.5204172	0.006	0.76
	*R*-squared	0.58		

*Y* left acetabulum axis (cos⁡Y_l)	Intercept	0.2052325		0.0
	(cos⁡Y_r)	0.3206353*E* − 03	0.002	0.81
	*R*-squared	0.67		

*Z* left acetabulum axis (cos⁡Z_l)	Intercept	0.790657		0.0
	Pelvic incidence	−2.888895*E* − 03	0.0071	0.75
	*R*-squared	0.567		

**Table 4:** The residuals (difference between measured and predicted *X*, *Y*, *Z* acetabulum axis) in each model and the confidence limits of predicted *X*, *Y*, *Z* acetabulum axis.

*n* = 12	*X* right	*Y* right	*Z* right	*X* left	*Y* left	*Z* left
Degree	Acetabulum axis	Acetabulum axis	Acetabulum axis	Acetabulum axis	Acetabulum axis	Acetabulum axis
Mean/SD	−0.05/4.98	−0.64/7.52	1.39/4.92	0.21/3.26	−0.47/4.79	1.18/2.97
Max/min	8.01/−8.47	13.93/−9.29	8.63/−4.93	3.65/−6.89	7.50/−6.57	6.28/−2.58
95% CL of mean	2.9	4.4	2.9	1.9	2.8	1.8
99% CL of mean	3.9	5.8	3.8	2.5	3.7	2.3
